# Supplementary material for: Depression and anxiety among quarantined population during the COVID-19 outbreak in central Ethiopia
Source: PLOS Glob Public Health. 2022 Jul 1;2(7):e0000115. doi: 10.1371/journal.pgph.0000115 (PMC10021643; doi:10.1371/journal.pgph.0000115)
Supplement: Attachment — Submitted filename: Point by point to ND PLOS ONE (revised Sep 11 2020).docx [file pgph.0000115.s002.docx]

**Point by point responses for reviewers comments**

Dear Cristian R. Montenegro, Academic Editor, PLOS GLOBAL PUBLIC HEALTH

Thank you very much for giving us the opportunity to revise our manuscript “Prevalence and correlates of depression and anxiety in quarantined population during the COVID-19 outbreak in central Ethiopia**”**.

We would also like to thank the reviewers for detailed reviews and providing us with helpful suggestions that will strengthen our manuscript and knowledge. We have gone through the comments and tried to include the responses to all the comments and suggestions.

**Comments by editor and reviewers**

**Editor’s comments**

Additional Editor Comments (if provided):

1) somewhere in the introduction, a timeline or reconstruction of key events in response to the pandemic in Ethiopia should be provided to help the reader better understand the context.

**Author reply:** Thank you. We addressed this concern accordingly. We have included the following paragraph in the introduction section sections” Even though the response to quarantine should include assistant for those in quarantine ─ this includes screening to identify risky people, coordinated and consistent provision of COVD-19 related knowledge and attitude, as well as basic psychological support to those in quarantine. However, this basic support for quarantine population is not undertaken in Ethiopia, which may increase the risk of mental health people in those population groups”. *(See page 4-5 background section line 58-63)*

2) The article calls for attention to be given to mitigate mental health problems in the quarantined population during the COVID- 19 outbreak. One would expect a more concrete, evidence-based kind of suggestion, differentiated along the lines of the specific results and not a generical "more attention" type. Attention to what specifically? What kind of interventions could be developed?

**Author response:** Thanks. We have now included the following sentence in the introduction section sections **“**These include identification support for those vulnerable groups and psychological support to reduce the stress associated adverse mental health outcome”. *(See page 5 background section line 70-71)*

**3) Careful proofreading is required.**

**Done.**

**Reviewer #1**

**Title**

 Many thanks for having the chance to review this article that aimed to explore the prevalence and factors associated with anxiety and depression among in quarantined population during the COVID-19 outbreak.
- Title should be shortened (including both prevalence and correlates of depression and anxiety) confuse the reader. Please be more specific.

***Author response:*** *Thank you. We have revised the title as “Depression and anxiety among quarantined population during the COVID-19 outbreak in central Ethiopia” (See title page)*

**Abstract**
- Background part is very large. Please shorten.

*Response: We have shortened the this section as follows: Evidence suggests that quarantine might have a wide-ranging, substantial, and long-lasting negative psychological impact especially when the necessary preventive measures are not taken. This study assessed the prevalence and associated factors of depression and anxiety among the quarantined population during the COVID-19 outbreak in central Ethiopia” (See page 2 abstract section )*

- At the methods part few things are repeated. Please reformulate.

**Done**
- At the conclusion part be more specific. Do not repeat the results section.

**Done.**

Introduction
- Please update information about Ethiopia

*Response: Thank you. We have included the following in the introduction section “In Ethiopia, the second populous nation in Africa (110 million people) as of January 14^th^, 2021, a total of 129,455 confirmed cases and 2,006 deaths have been reported”. (See page 3 background section line 14-16)*
- Paragraph 1 and 2 should be merged.

*Author reply: Thank you. We have revised and merged aparagrpah1, 2, and 3 to avoid repetitions.*
- Paragraph 3 second sentence. You are saying about quarantine and after jumping directly to infectious out

brakes. An infection outbreak doesn’t mean quarantine so please clarify this part.

This has been revised accordingly.

- In general, the introduction part is very long. Similar things are repeated. Is better to shorten this part and to be more concise on what you want to say.

Revised and shortened as suggested.

**Methods**

- Subsections at the methods part should be more concrete and no so general (i.e. Study population, sample size determination, sampling technique, and inclusion criteria). Please split these parts.

*Response: Thank you.* ***Done****.*

- Please give more information / clarify about “purposively selected the quarantine centers”.

- Its not very clear to me how researchers selected their sample;
*Author reply: This has been revised accordingly as follows “Regarding the sampling technique, first, we purposively selected and included all quarantine centers found in Addis Ababa, central Ethiopia (quarantine centers outside the capital city, Addis Ababa were not included). A simple random sampling technique was employed in order to select a representative sample of participants from the centers. The room numbers and the number of people at each center were taken from the responsible authorities handling this condition and control the overall situation of the quarantine centers ─ such as [Ethiopian Public Health Institute (EPHI) & the Federal Ministry of Health (FMOH)]. Then, we used a lottery method to select individuals from each center”. (See page 6 method section line 97-104)*

- Did you conduct face validity for questions of socio-demographic characteristics?

No, we did not.

- How were the researchers recruited?

- Who trained the researchers?
- Researchers were in pairs or individually during the process of data collection. Please give more information about this.

Response: Thank you. *Data collectors who have had prior experience and background in health sciences were involved (BSc in psychiatry/BSC in nursing). They were selected and trained by the principal investigator.* *(See page 7 background section line 120-126)*

- It is not clear to me something. You used a structured questionnaire. Why you needed to have trained researchers for this and especially so many.

*Response: We provide the training to make sure that all the data collectors and supervisors have clearly understood the questionnaire.*

- What about rejection rates? Which were the reasons for this?

*A total of 297 participants were included in the study. The response rate was 90.8%. (See page 8 result section line 147)*

- Participants in quarantine. How many of them were infected? Did you have differences between them infected and non-infected?

*We did not collect this information.*

- There is not the protocol number at the ethical approvement decision;

Response: We have included now.

**Results**
- First sentence of the results section. The whole sentence is one paragraph. Please split it.
- What do this sentence mean (second sentence of results part). Please clarify in US dollars. “Also, 209 (70.4%) had 8000 Ethiopian birrs and below as a monthly income with a mean of 8783.74.

*Author response: Thank you. We have revised this section accordingly. Here is the revised paragraph “A total of 297 participants were included in the study. The response rate was 90.8%. The mean age of the respondents was 28.33(SD=6.22) years. More than one-third of the participants (38.6%) were young adults (ages 18- 25 years). Approximately one-fourth of the participants were females (73.1%) and more than half were unmarried (56.9%) and Muslims by religion (51.5%). About 40.1% of the participant had attended primary education and the average income for the participants was 8783.74 Ethiopian birr (251.44 US dollars).* *Almost all the participants have been living abroad (99.3%).* ***(Table 1)***”. *(See page 8 result section line 147-155)*
- Please correct the first sentence (put absolute numbers in parenthesis) of the section Clinical and psychosocial characteristics;
- As above on the next section. Please check this in the whole text;
- How are defined the stressful events;
- In general sentences are very long. Please split them because the reader loses the meaning;
- The four first sections of the results part could be merged. Two many information are provided that are also available in the tables;

*Response: This section has been revised as “The majority of the participant (90.9 %) had a chronic illness and more than half spend their time on social media at the quarantine center (53.5%). Roughly half of the participants (49.8%) had intermediate social support and more than half (58.2%) had no stressful life events in the last 6 months. Regarding substance use history, approximately one-fifth of the participants had used tobacco (21.2%) and alcohol (18.2%) in their lifetime* ***(Table 2)****” (See page 8 result section line 157-161).*

- A table about bivariate analysis is needed;
- Is better to have in one table the adjusted and non-adjusted ratio rates;
- TO my view the focus should be on the multivariate analysis and not so much on the univariate. Too many information are given for the second but not the first.

*Author response: Thank you. The details of bivariate and multivariate analysis have been found in Tables 4 and 5.*

*We also revised the bivariate analysis section as follows for clarification*: *In bivariate analysis, socio-demographic factors such as having a child, stressful life events, spending the quarantine time by sleeping, and perceiving transmission of COVID-19 can be prevented by wearing of glove for anxiety were found to be significantly associated with anxiety in quarantine people* ***(Table 4)****. Whereas, marital status, stressful life event, method of refreshment, source of information about COVID-19, perception on the cause of COVID-19, and knowledge on method of prevention and utilization of COVID-19 prevention methods for depression were found to be significantly associated with depression among the participants****. (Table 5).*** *(See page 9 result section line 177-184)*
 **Discussion**

- Please clarify this “Sub-Saharan Africa including Ethiopia. Wasn’t the study conducted only in Ethiopia and more specifically in the capital.
- There are many other articles that says the opposite. “receiving different refreshing information and games through these social media’s as well as mass media’s could help the individuals to experience more relaxation and exposure with others. Please check this part.

*Author reply: This sentence has been revised as “This work represents one of the few studies that explored the prevalence and factors associated with anxiety and depression among in quarantined population during the COVID-19 outbreak in Sub-Saharan Africa including Ethiopia*”. *(See page 9 result section line 197-199)*.

- Last paragraph of the discussions is similar to the third paragraph. Please check.

*Response: Thank you. We have double-checked this. They are different ─ stressful events were linked with both anxiety and depression.*

- Which are the strengths and limitations of this study?

*Author reply: Thank you. We have addressed this concern accordingly. We have included the following in the discussions section: (See page 12 result section line 273-280)*.

***Strength and limitation of the study***

*The current study had several strengths: First, large sample size and it is one of the few studies on the subject. Second, we have used standard instruments to measure anxiety and depressive symptoms.*

*The limitations include: due to the nature of the study (cross-sectional study design) the association between the different variables with anxiety and depression may not imply causality. Recall bias is another limitation of the study since we have retrospectively assessed most exposure variables including a history of chronic medical illness and substance use problems.*
**Conclusions**
*- Please mention implication for future research.*

*Response: We have included the following in the conclusion section “The results suggest that attention needs to be given to mitigate mental health problems in the quarantined population during the COVID-19 outbreak. Screening for mental health issues and providing mental health (psychological) support are needed for the quarantine population. (See page 12-13 result section line 288-292)*.

- English editing is needed for the whole text.

**Done.**

Reviewer #2: This is an important paper, being one of the first studies on the effects of quarantine on mental health in the Global South. The findings are partially expected, but leave some questions unanswered that need to be addressed. Also, methods and data analysis should be further fleshed out as noted below.

First, it is unclear how exactly quarantines work in Ethiopia. For example, what is the criteria to determine someone should quarantine? Testing positive for COVID-19? Having a close one who tested positive for COVID-19? Entering the country? All of them? That should be clearly stated so we know who the target population is.
Also, just wondering, did the government implement any quarantines affecting entire neighborhoods/communities? Were people from those communities also of interest to this study? Were those “total quarantines” or “partial quarantines” (e.g., only for the weekends)? You may want to operationalize what “being in quarantine” means here.

*Author response: Thank you. During the study period, people who enter the country need to enter into 14 days mandatory hotel quarantine following arrival. We including the following in the method section for clarification (See page 5 method section line 83-87)*.

*“During the study (July 2021), Ethiopia has implemented 14 days of mandatory hotel quarantine for all travelers who are permitted to cross the border. (*

***Study population***

*The study population included all individuals under quarantine who were included in the sample.”*

Second, the description of the target population is somewhat incomplete. Who were these people? How different were they compared to the general population? Were they at higher risk of reporting mental anxiety/depression? All this should be said explicitely (e.g., as hypotheses). The authors should note, though, that given we don’t know much about this population it’s challenging to say that they were at higher risk of mental symptoms. For instance, we don’t know if they had a prior mental/physical condition or if their parents had mental/physical conditions too, both of them strong predictors of mental issues.

**Response:** Almost all participants were international arrivals who were under mandatory hotel quarantine (99.3%). As mentioned in the results section (table 1) almost all the participants have been living abroad.

See below the sentence included in the method and result section for clarification.

*Methods (See page 5 method section line 85-87)*.

***Study population***

*The study population included all individuals under quarantine who were included in the sample.”*

*Result (See page 8 result section line 154-155)*.

*Almost all the participants have been living abroad (99.3%).*

Third, it seems the authors used the HADS plus a series of ad-hoc questions on, for example, social support, stressful life events, substance use history, etc. Please describe those questions/items at length. Furthermore, why did the authors collect data on those variables in particular? Were those hypothesized to be related to the exposure and the outcome (i.e., confounders)? If so, it should be mentioned in the Introduction/Background.

*Author response: Thank you. We have included factors that have been associated with depression and anxiety in quarantine people in prior studies. (See page 6-7 method section line 105-126)*.

*We have clarified the method section as follows: A structured questionnaire containing data on socio-demographic characteristics (age, sex, and marital status), quarantine and clinical factors, psycho-social and substance-related factors was used to collect information from the participants. These were relevant factors that have been repeatedly linked with anxiety and depression in quarantine people in previous studies.*

*We also included the following statement in the method section: Social support was measured by Oslo on the 3 items social support scale (OSS-3), which has been commonly used to assess social support and had been used in several studies(25,26). It has a score ranging from 3-14, which had three broad categories: “Poor Support” 3-8, “Moderate Support” 9-11, and “Strong Support” 12-14.*

Fourth, the data analytic plan needs refinement. For example, how did the authors conceptualize the exposure/predictors, outcome, confounders, etc? It seems they put everything into a logistic model and saw what came up significant. This approach has been largely criticized in epidemiology because some of those variables could be considered indeed as confounders, but others could be effect modifiers and/or mediators (for which you don’t have to control for). Please provide a rationale for your analytic approach taking into consideration all this.

*Author reply: Thank you. We have considered P-value and Braford Hill’s criteria to sect important variables.*

*We have included the following in the method section “Bivariate and multivariate analyses were performed to determine the presence of statistically significant associations between the independent and dependent variables. Those variables with a p-value <0.02 in the bivariate analysis were selected to be included in the multivariable logistic regression to control for possible confounders. Additionally, we have used Bradford Hill’s causal criteria including the consistency of the association to the select important variables to include in the multivariable model (27”. (See page 7 method section line 131-138)*.

Fifth, the authors should distinguish between “crude” versus “adjusted” results, and say which variables they adjusted for (e.g., sociodemographic?). Also, please describe variables of interest before presenting the results. For example, the following paragraphs makes no sense if you haven’t defined each variable previously: “bivariate analysis was done and socio-demographic factors such as having a child, stressful life event, spending the quarantine time by sleeping, and perceiving transmission of COVID-19 can be prevented by wearing of glove for anxiety and factors such as marital status, stressful life event, method of refreshment, source of information about COVID- 19, perception on the cause of COVID-19, knowledge on method of prevention and utilization of COVID-19”

*Response: We have addressed these comments accordingly.*

*We have revised the bivariate analysis section as follows for clarification: In bivariate analysis, socio-demographic factors such as having a child, stressful life events, spending the quarantine time by sleeping, and perceiving transmission of COVID-19 can be prevented by wearing of glove for anxiety were found to be significantly associated with anxiety in quarantine people* ***(Table 4)****. Whereas, marital status, stressful life event, method of refreshment, source of information about COVID-19, perception on the cause of COVID-19, and knowledge on method of prevention and utilization of COVID-19 prevention methods for depression were found to be significantly associated with depression among the participants****. (Table 5)****. (See page 9 result section line 177-184)*
