# Supplementary material for: Depression and anxiety among quarantined population during the COVID-19 outbreak in central Ethiopia
Source: PLOS Glob Public Health. 2022 Jul 1;2(7):e0000115. doi: 10.1371/journal.pgph.0000115 (PMC10021643; doi:10.1371/journal.pgph.0000115)
Supplement: Attachment — Submitted filename: Point by point to ND PGH NOV 021 R2.docx [file pgph.0000115.s003.docx]

**Point by point responses for reviewers comments**

Dear Cristian R. Montenegro, Academic Editor, PLOS GLOBAL PUBLIC HEALTH

Thank you very much for giving us the opportunity to re-revise our manuscript **Depression and anxiety among quarantined population during the COVID-19 outbreak in central Ethiopia”**.

We would also like to thank the reviewers for detailed reviews and providing us with helpful suggestions that will strengthen our manuscript and knowledge. We have gone through the comments and tried to include the responses to all the comments and suggestions.

**Comments by editor and reviewers**

**Editor’s comments**

**Additional Editor Comments (if provided):**

1) Check references

**Author reply:** Thank you. We addressed this concern accordingly. Changes made are highlighted in yellow─reference 25 and 40.

2)  **Careful proofreading is required.**

**Done.**
